# Removing financial barriers to access reproductive, maternal and newborn health services: the challenges and policy implications for human resources for health

**DOI:** 10.1186/1478-4491-11-46

**Published:** 2013-09-22

**Authors:** Barbara McPake, Sophie Witter, Tim Ensor, Suzanne Fustukian, David Newlands, Tim Martineau, Yotamu Chirwa

**Affiliations:** 1 Institute for International Health and Development, Queen Margaret University, Edinburgh, UK; 2 Nuffield Institute for Global Health, University of Leeds, Leeds, UK; 3 Department of Economics, University of Aberdeen, Aberdeen, UK; 4 Liverpool School of Tropical Medicine, Liverpool, UK; 5 Biomedical Research and Training Institute, Harare, Zimbabwe

**Keywords:** User fees, Human resources for health, Policy co-ordination, Distribution, Workload, Pay

## Abstract

**Background:**

The last decade has seen widespread retreat from user fees with the intention to reduce financial constraints to users in accessing health care and in particular improving access to reproductive, maternal and newborn health services. This has had important benefits in reducing financial barriers to access in a number of settings. If the policies work as intended, service utilization rates increase. However this increases workloads for health staff and at the same time, the loss of user fee revenues can imply that health workers lose bonuses or allowances, or that it becomes more difficult to ensure uninterrupted supplies of health care inputs.

This research aimed to assess how policies reducing demand-side barriers to access to health care have affected service delivery with a particular focus on human resources for health.

**Methods:**

We undertook case studies in five countries (Ghana, Nepal, Sierra Leone, Zambia and Zimbabwe). In each we reviewed financing and HRH policies, considered the impact financing policy change had made on health service utilization rates, analysed the distribution of health staff and their actual and potential workloads, and compared remuneration terms in the public sectors.

**Results:**

We question a number of common assumptions about the financing and human resource inter-relationships. The impact of fee removal on utilization levels is mostly not sustained or supported by all the evidence. Shortages of human resources for health at the national level are not universal; maldistribution within countries is the greater problem. Low salaries are not universal; most of the countries pay health workers well by national benchmarks.

**Conclusions:**

The interconnectedness between user fee policy and HRH situations proves difficult to assess. Many policies have been changing over the relevant period, some clearly and others possibly in response to problems identified associated with financing policy change. Other relevant variables have also changed.

However, as is now well-recognised in the user fee literature, co-ordination of health financing and human resource policies is essential. This appears less well recognised in the human resources literature. This coordination involves considering user charges, resource availability at health facility level, health worker pay, terms and conditions, and recruitment in tandem. All these policies need to be effectively monitored in their processes as well as outcomes, but sufficient data are not collected for this purpose.

## Introduction

Universal health coverage (UHC) has been labelled, 'the most powerful unifying single concept that public health has to offer’, by Margaret Chan, Director of the World Health Organization [1].

UHC has become an international policy. In 2007, universal access to reproductive health was included among the Millennium Development Goals (MDG, goal 5b), which were unanimously agreed by all UN member states as part of the Millennium Declaration [2]. A UN resolution was passed by the UN General Assembly in December 2012, urging governments to ensure whole population access to affordable, quality, health services^a^. The primary initial focus of UHC policies has been on the extension of financial protection against health care costs through the provision of insurance and the removal of user fees at the point of use.

Specific international attention to sexual and reproductive health has placed such services at the forefront of the UHC debate. A total of 179 nations committed to protect reproductive and health rights of women and girls at the International Conference on Population and Development (ICPD) in Cairo in 1994, and this was reaffirmed at subsequent conferences in Beijing and Copenhagen. The High-Level Task Force for ICPD was established in 2012 to take forward this agenda in the period leading up to the twentieth anniversary of this commitment^b^. Inclusion of MDG goal 5b was owed to the momentum of these conferences [2]. Consequently, Reproductive Maternal and Newborn Health (RMNH) services have often been the first priority of the UHC policies of the different countries.

With respect to removal of fees, this has amounted to a paradigm shift, with a growing consensus that user fees are regressive and undermine equitable access to essential health services [3], as do all types of out-of-pocket payment [4]. In particular, a concern that pregnant women and children under five years are negatively affected by such financial barriers has prompted many low- and middle-income countries to reconsider levying user charges by ensuring either more thorough implementation of exemption or waiver mechanisms, significant reduction in fee levels, or their abolition altogether [5,6]. While its impact on the level of out-of-pocket payments in the health system may not be definitive (and indeed, most health systems remain heavily dependent on out-of-pocket payments), such a policy shift [5] will undoubtedly have consequences for the health system across a number of dimensions, including the search for replacement revenue and ensuring quality in responding to the changes in utilization, reflecting increased numbers and patterns of utilization [3,7]. Both of these anticipated consequences raise specific concerns for human resources for health (HRH), yet this issue has been frequently overlooked until recently. Campbell *et al*. [5] suggest that demand-side support, ensuring that access is no longer constrained by payment for services, requires balance with support for the supply side in terms of capacity and quality of care. This research responds to this concern.

The objective of this research was to determine the associations and inter-relationships between workforce characteristics (stock, distribution and competencies) and equitable access to RMNH services resulting from the removal of, or exemption from user fees.

The research questions that we sought to answer were to understand the evidence of the impact of fees, exemptions and fee removal on HRH, and of HRH characteristics on the impact of fees, exemptions and fee removal. The sub-questions, to be addressed specifically in Sierra Leone, Zimbabwe, Zambia, Nepal and Ghana were: to describe the RMNH workforce in terms of its stock, distribution, skill mix, workload, remuneration and terms and conditions; to project need for RMNH workforce and identify plans in place to enhance quantitative and qualitative capacity; to describe the situation with respect to formal fees and exemptions, the revenue generated and its distribution, and effects on demand for health care; and to identify the policy implications.

## Background

In the five case-study countries, there have been significant developments in both financing and human resources for health policy that have led to their selection as case studies and provide the background to the study. In Ghana, Nepal, Sierra Leone and Zambia, the health system was designed during the mid-twentieth century or earlier to provide universal coverage through a public health-care system that is free at the point of use, financed largely through the government budget and mainly, therefore, through taxation and funds derived from development assistance. In the post-independence period, problems emerged to varying degrees, in sustaining accessible services at an adequate level of quality through this mechanism, and were generally attributed to funding shortfalls. User charges started to be introduced as early as 1969 in Ghana and as late as the early 1990s in Zambia. In Zimbabwe, fees existed at independence, but the exemption system effectively qualified most families for free health care until the mid-1990s when similar pressures emerged and user fees increased and became more widely applied.

In Ghana, exemptions were introduced for delivery care in 2004, first in five regions, then across the country. The policy was later superseded in 2008 by free coverage of all pregnant women within the National Health Insurance Scheme (NHIS). Both policies were undermined by poor availability of funds. Government HRH policy focused on task shifting and improving distribution, including introducing the deprived area incentive scheme, augmenting salaries in 55 districts. Large pay increases were funded in 2006 and in 2006 to 2007 there was a significant expansion of training schools, although there were also some concerns about the effect of this expansion on quality of training.

In Nepal, in principle, all citizens have free access to primary care. Targeted groups are also protected from secondary care costs. However, both policies have been undermined by shortfalls in funding. In 2005, financial incentives were introduced to encourage women to deliver in a facility, and in 2008 the *Aama* policy was introduced, providing free institutional deliveries in all public and some private facilities. The HRH strategy of 2003 aimed to increase the public sector workforce by 71% by 2017, with an emphasis on ensuring increased numbers of health workers with skilled birth attendance competencies.

In Sierra Leone, the Free Health Care Policy (FHCP) was introduced in 2010, providing for free public care for pregnant women, lactating women and children under five years. Substantial salary increases were funded in 2011 and a performance-based financing system at district level was introduced in 2011. HRH policy planned incentives for hard-to-reach areas and reformed career paths and recruitment processes, although little progress had been made in these areas at the time of the research.

In Zambia, user fees were abolished for rural primary care in 2006 and in peri-urban areas in 2007 in both government and mission facilities. A Department for International Development (DFID) grant was provided to enable compensation for the resulting loss of revenue. HRH policies emphasized the training and recruitment of graduates, the development of a human resource (HR) information system, and the scaling up of the Zambia Health Workers Retention Scheme, offering salary top-ups in remote areas.

In Zimbabwe, there was a policy of free care but it had been inconsistently applied, and there was a perception that charging could be locally determined. HRH expenditure collapsed to 0.3% of the public health budget in 2008. Dollarization of the economy may have improved the position of health workers since then, and also increased the real value of those fees that are charged. An Emergency Retention Scheme was introduced, supporting salaries of key professional cadres, but this will be phased out by 2013. The HRH strategic plan identified the key priority of staff retention.

## Methods

This study consisted of the following components: literature review, desk-based analysis and document review, field studies and analysis. No experimental research or research on humans was involved in this work.

### Literature review

We undertook a review of the current literature on the removal of, exemption from or waivers of user fees in low- and middle-income countries in relation to RMNH and the consequences for human resources for health working in RMNH. First, to be included, studies had to address either the removal of user charges or the application of exemptions and/or waivers in order to facilitate access to RMNH services in low- and middle-income countries. The user fee, exemption and waiver mechanisms at national, provincial and district level were explored. The second criterion for inclusion was consideration of the effect of these financing instruments on RMNH health personnel, particularly cadres of skilled birth attendants (SBAs), including nurses, midwives, doctors and clinical officers and the paramedical, support and ancillary staff.

The final criterion was publication date, which was restricted to 2001 to 2011, with some exceptions, where studies on the introduction of user fees from the 1980s to 1990s were included for historical context. Only studies and reports written in the English language were collected, collated and consolidated in the bibliography. The following databases and sources were searched: PubMed, Popline, SCOPUS, Science Direct, Web of Knowledge, Human Resources for Health Journal, Equinet, MNCH knowledge portal, ELDIS, HRH Global Resource Centre, World Health Organization, Alliance for Health Policy and Health Systems Research, and Google Scholar, using a list of 66 keywords.

In the initial search, 500 articles were identified, out of which 267 were shortlisted based on the keywords above; the abstracts were then reviewed independently by two researchers and 115 were shortlisted. Following a further refinement of the search parameters, in which the keywords were narrowed to exclude any articles not including reference to human resources engaged with RMNH activity, a final list of 67 was included and the full articles were included and reviewed. Similarly, the grey literature search furnished 200 documents and 35 were included following the aforesaid procedure.

### Desk-based data analysis and document review

We sought data on:

• Human resource numbers and distribution (by cadre and district) in public and private sectors and before and after the financing policy change of interest, where relevant;

• Public and private sector remuneration and allowances, and trends;

• RNMH need as measured by the population and birth rate by district;

• Health-management information-system data on levels of use of antenatal care, postnatal care, deliveries, newborn care, abortions, and family planning, gynaecological, sexually transmitted diseases (STD) and HIV clinic services.

Access to data sets held by Ministries of Health, Central Statistical Offices and similar offices was secured along with policy and planning documents, through the recruitment of local collaborators in a position to access these. Grey literature was located by web search and by contacting relevant local agencies. The search for data and documents was undertaken during 2011.

Much of the data sought proved unavailable. Trend data were generally unavailable either due to an absence of maintenance of a historic database, or because previous estimates of variables were made in a way not comparable with those of present estimates. Private sector data were difficult to access and sparse where available at all.

### Field studies

Field studies were undertaken in two countries (Sierra Leone and Zimbabwe) to gain more in-depth understanding in both HRH and financing domains. These countries were selected because there was a smaller literature base on user fees and their removal, in these countries than in others. In Sierra Leone, the time was spent accessing documents and secondary data and seeking clarifications in relation to data that appeared inconsistent. Data quality was poor, and there remain considerable gaps in what we were able to collect.

### Analysis

In each country we analysed available data and research reports to review: (1) how financing policy change had affected utilization levels; (2) the geographical distribution of the health workforce; (3) delivery workloads and how actual workloads and potential workloads (based on the total number of births that are estimated for the country) compared to what is considered by the WHO to be a feasible workload; and (4) remuneration and terms and conditions. In the discussion section, we address to what extent a review of these data help to answer our research questions concerning the inter-relationships between workforce and financing situations and policies.

Qualitative data were transcribed and analysed thematically, starting from the topics outlined in the interview guides, but allowing for identification of new themes arising from the discussions. Analysis of the distribution of the health workforce in each country computed concentration indices (CIs). These are constructed by ordering districts by increasing population density (from most sparsely to most densely populated districts) and measuring the distance between actual and equal shares of health workers per head of population in each district. A hypothetical situation where health workers are distributed equally in proportion to population across the country produces a CI of zero (no distance from actual to equal share). In a situation where the distribution favours densely populated areas, the index will be greater than zero. Maximum, pro-urban, concentration is where the whole of the staff is based in the most densely populated district and the corresponding CI is one.

## Literature review

In the mid-1980s, many low- and middle-income countries were encouraged to introduce user fees as a response to declining national health budgets. User fees were presented as a means of cost recovery of public health expenditure, as well as enhancing efficiency and equity [3,8]. The Bamako Initiative, put into action by African Ministers of Health, followed on closely in 1987. It included user fees among its instruments, amid assertions that it would produce quality improvements in services through the local retention of generated revenue. It also placed a strong emphasis on community participation [7,8].

However, after more than two decades of global user-fee experience, these objectives have been rigorously critiqued [9-11]. As the regressive nature of user fees has come under close scrutiny [3,9,12] many countries have taken steps to either reduce or abolish user fees in their health facilities, or to more consistently apply exemptions or waivers from fees for specific groups or services [13-15].

The slow progress in reducing the high levels of maternal and neonatal deaths in low income countries has led to a renewed commitment to improve provision and access to RMNH services [3]. At least three quarters of neonatal deaths and a similar proportion of maternal deaths occur outside hospital [16]. User fees are cited as a considerable financial barrier to women’s care-seeking during and following their pregnancy [17,18]. A long list of countries including Benin, Burkina Faso, Burundi, Cameroon, Ethiopia, Ghana, Kenya, Liberia, Mali, Nepal, Niger, Senegal, South Africa, Sudan, Uganda and Zambia have pursued fee removal or exemption policies for delivery care and/or caesarean section [6]. Most studies reviewing utilization following the abolition of user charges for deliveries and other related maternity care have observed a rise in assisted deliveries and caesarean sections at health facilities [3,13,18-25] and, in some cases, show that gains are concentrated in poorer groups [8,14].

Campbell *et al*. [5], however, present another perspective in acknowledging the challenges now presented by fee removal:

What is the net benefit of increasing access to 'free’ health services if there is no qualified health worker available to provide care, or where you may queue all day only to be afforded an ineffectual consultation which undermines respect, trust, privacy and confidentiality? Such are the realities in many low-income countries, particularly in rural and remote areas, where health workers are drastically in short supply, and often over-burdened and ⁄ or under-resourced (p.1)

Lee *et al*. [17] concur, arguing that, 'strategies to increase demand for services need to be accompanied by actions to ensure the supply side can cope with the increased demandʼ (p.114).

Recent reviews of the growing trend to abolish or suspend user fees highlight that for these policies to be effective, careful planning of the supply-side response to the stimulated demand has to take place [8,9,13,23,24,26,27].

The literature generally underplays the important contribution of fee revenue at facility level [27]. In Senegal, for example, at the higher levels of the system, user fees made up 37% of the revenue of the regional hospital and 43% of the *Centres de Santé*, whereas the health posts derived 95 to 96% of their revenues from user fees [21]. In situations where fee revenue was retained by the district or sub-district facility, it also allowed some autonomy and flexibility for the district health management team or the health centre in charge to respond to gaps in funding [28]. Such discretionary funding would often supplement low salaries, cover delays in receiving salaries or cover the costs of community or support staff [21,24,28-30]. Several studies, for example, Kipp *et al*. [31], also describe the important role such incentives had on staff motivation. Often, technical and community support staff received wages or small bonuses from user-fee revenues in Afghanistan [28], Uganda [32] Zambia [29] and Senegal [21].

Increases in utilization lead to increases in staff workloads if there is no additional recruitment. In several countries this was anticipated with a concomitant rise in salary; in other countries a lack of preparation and planning compounded the problem of staff shortages and difficulties with rural allocation and retention, leading to significantly low morale.

Most studies reported that health staff considered their workload to have increased since the new policies on fee removal or exemptions commenced [13,19,24,28,33,34]. Witter *et al*. [21] report similar increases (of about one third) in delivery workloads for midwives in Senegal and medical assistants in Ghana [18,22]. Concomitant with increased workload, various studies report declining morale - in Burundi [33], South Africa [190], and Uganda [24] - made worse where allowances or bonuses are also removed, as in Zambia [29]. In both Zambia [29,35] and Uganda [36-38] additional funding was released by the Ministries of Health to the districts to compensate for loss of revenue. In Uganda, according to Nabyonga-Orem *et al*. [36], flexibility in how these funds were to be used was allowed, although Ssengooba *et al*. [30] suggest that the additional funds did not directly compensate staff. In Zambia, few guidelines were provided by the Ministry of Health about what the 'user fee replacement grants’ could be used for [29], decisions about their use were centralized and distribution did not reflect the former levels of user-fee collection [39].

Loss of financial autonomy provided by user fees has been regretted in a number of countries, including Burundi. Before the introduction in 2006 of free health services for children under five years and free deliveries, hospitals retained all user fees and were expected to be relatively self sufficient [33]. Following the abolition of fees for these services, delays in reimbursement affected hospital and health-centre functioning, as they now could not pay for their own supplies. In Burkina Faso, the 20% bonus formerly received by RMNH staff for deliveries from the user fee was retained but with no guidance on how to calculate from which price they should take the 20% [13,26].

In Nepal and Burkina Faso, reimbursement tariffs were decided centrally by the Ministry of Health. Nepal’s national free delivery policy has retained incentive payments to health workers of the earlier scheme [14]. The tariffs in Nepal varied according to facility type and degree of obstetric complication [15]. In Niger, the additional administrative and clinical workload experienced by health workers, and created by increased utilization, was acknowledged, and a payment of a monthly bonus supplemented their salary [40].

Community and support staff often had to be made redundant once facilities no longer had discretionary funds from fee revenue. This occurred in Uganda [32]. Some of the staff of the *Centres de Santé* community in Senegal received a fixed monthly allowance [21].

Very few studies identified the cadres affected by fee removal or exemptions. Witter *et al*. [21] cite a shortage of midwives in Senegal. The workforce associated with delivery care in Nepal remained stable or increased, but increases were not directly related to financing policy [14] (p.89).

While Hoope-Bender *et al*. [41] argue that, 'most primary health care frontline workers are not sufficiently skilled to deliver a minimum MNH service packageʼ (p.230), others are more hopeful that a process of careful planning for task shifting could produce sufficient skills in lower cadres to meet the need, including performing caesarean sections [42].

## Case studies

### How financing policy change affected utilization levels

In Ghana, the delivery exemption policy appeared to be effective in raising utilization with some modest equity gains [18]. One study has compared baseline data in two districts, before the NHIS (in 2004) and after (in 2007) [43]. Its findings suggest that there has been an increase in access to formal care amongst members, as well as a significant decrease in out-of-pocket expenditure. However, there was no difference in use of maternal care (antenatal care (ANC), deliveries or caesarean sections) between the intervention and control group.

While there is no public information on trends in use of outpatient services by insured patients specifically, outpatient use for the population as a whole shows a marked increase from 2005 onward, compared to stable (low) use before. The timing and pattern correlated with growth in NHIS membership, indicating that the NHIS has indeed increased service use [44]. According to an International Labour Organization (ILO) paper of 2006, 'utilization for the insured was then at around 0.9 (OPD [out patients] per capita - almost twice the non-insured (then at 0.49 visits per capita)’ [44]. However, even the rate for the insured falls far below the Service Availability and Readiness Assessment (SARA)^c^ benchmark level of 5 [45]. It is also interesting to note that overall admissions have not experienced consistent growth between 2005 and 2008. This might reflect the benefits of early intervention through better access to outpatient care.

In Nepal, the latest household survey on the *Aama* programme [46] indicates that over the past five years, there has been a substantial increase in the proportion of women giving birth in a health facility (albeit from very low levels). In high Human Development Index (HDI) districts, the rate of institutional delivery care has increased from 33 to 54% and in low HDI districts from 6 to 21% between 2005 and 2010.

There was some evidence of pro-poor impact of fee exemption: three low-HDI districts saw higher rates of free-facility births than three high-HDI districts, and in the low-HDI districts, poorer women were more likely to receive free care. Trends over time by wealth group show that inequality in facility births has fallen substantially and marginalized castes have seen large increases in utilization over the past five years.

In Sierra Leone, the impact of the FHCP on utilization appears mixed [47]. For outpatient visits of children under five years, there was a more than twofold increase in the number of consultations in the twelve months post-FHCP introduction compared to the last year before the FHCP. However, this conceals a gradual downward trend in the later part of the first year post-FHCP, and even after the increase there were fewer than 0.5 consultations per member of the population per year. Liaqat and Ferry [48] confirm that there was a sharp and statistically significant increase in health utilization by children under five years across Bombali District immediately after the introduction of the FHCP, but the peak was not sustained. In the immunization of children under age one, 88% of children were fully immunized pre-FHCP but this had fallen to 76% post-FHCP [47].

In maternal health, there was an increase of 45% in the number of pregnant women making at least one ANC visit. There was an initial increase in the number of postnatal care (PNC) consultations, but a slight reduction towards the end of the first year. The number of new acceptors of modern family-planning methods increased by about 140% in the first 12 months of the FHCP [49]. Again, these percentage increases must be understood in the context of very low initial levels of service use.

In Zambia, after free care was introduced in 2006, an analysis of facility records from the Health Management Information System (HMIS) showed that removing user fees for primary health care services increased the number of outpatient visits in rural districts by patients over five years of age, and achieved visit-per-capita rates of two in rural districts, well above the African and urban Zambian average, if still far below SARA^d^ benchmarks [35,50]. However, there was a wide difference across districts, ranging from a fall of 39% to an increase of more than 100% [51]. The increase in utilization was not always sustained over time and there was indication of crowding-out of children under five years, who already received care free of charge before the policy change. Analysis of a comprehensive national facility-based dataset found that utilization increased by 55% among the rural population aged at least five years. Utilization increases were greatest in the districts with the highest levels of poverty and material deprivation [35], although this analysis regressed percentage utilization change with district deprivation score, and may have been confounded by an underlying correlation of initial utilization levels and deprivation. However, analysis of the Living Conditions Measurement Surveys (LCMS) did not confirm an increase in access to care. The analysis found no evidence that removing fees improved the probability to seek care when falling ill, even after adjusting for the varying degree of implementation of the policy across districts [51].

In Zimbabwe, there is a lack of clarity about the levels of fees that should apply, and have, in practice, applied over recent decades. This implies that analyses of utilization trends cannot be linked effectively to discrete changes in policy and implementation. However, there is evidence that fees act as a deterrent to use of health care [49].

In the interviews we conducted, delivery care in facilities was viewed as difficult to afford for most families, even in the absence of complications. This is likely to be one factor behind the high rate of home deliveries, even though these are discouraged (and as a result, traditional birth attendants (TBAs) were nervous about speaking about their work), and despite the fact that families have to bring newborn babies into health facilities to get a birth record [52].

### The geographical distribution of the health workforce

WHO defines an SBA as,

… an accredited health professional – such as a midwife, doctor or nurse – who has been educated and trained to proficiency in the skills needed to manage normal (uncomplicated) pregnancies, childbirth and the immediate postnatal period, and in the identification, management and referral of complications in women and newborns^e^.

While this definition is clear in principle, it is not always easy to operationalize in any given context. Some categories of staff in use include those who have and have not been educated in this set of competencies. For example, some nurses may have undertaken specialist childbirth-related training and some not. Furthermore, those whose training has included these competencies may not have undertaken relevant practice in the meantime, or may not have retained them for other reasons. The categories of staff, at least some of whom have SBA capacities in the five countries, can be listed as doctors, nurses, midwives (not a separate category from nurses in Nepal and Zimbabwe), clinical officers (Zambia and Zimbabwe only), and auxiliary nurse midwives (Nepal only).

We were able to disaggregate data for public and private sectors in Ghana, Nepal and Sierra Leone, and further separate non-governmental organizations (NGOs) and faith-based organizations (FBOs) from the rest of the private sector in Sierra Leone. These data show that in Ghana, most health professionals work in the public sector, namely, 80% of doctors, 80% of midwives and 91% of nurses. In Nepal the situation is almost reversed: 83% of doctors, 59% of nurses and 33% of auxiliary nurse midwives work in the private sector. In Sierra Leone, 58% of doctors, 62% of nurses and 66% of midwives work in the public sector while 15% of doctors, 16% of nurses and 16% of midwives work in FBOs. The remainder work in the for-profit sector. In Zambia it is reported elsewhere that 80% of health workers worked in the public sector in 2006 [53] and in Zimbabwe, it has been estimated that 45% of doctors work full-time in the private sector [54].

**Figure 1:**
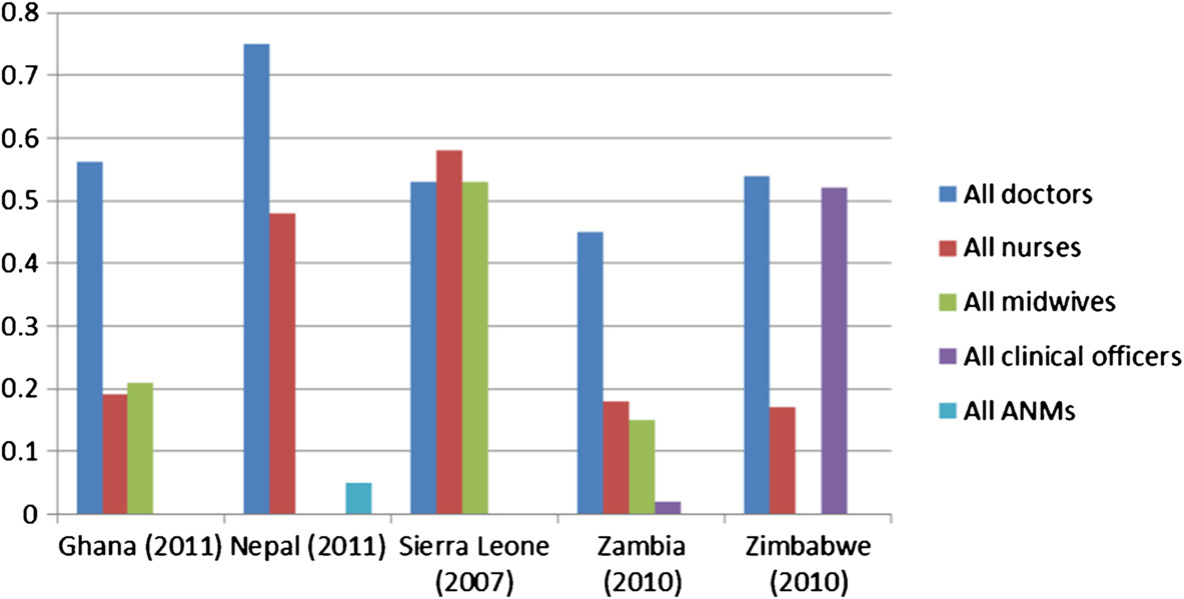
**Concentration indices**: **health workers by cadre**, **latest available dates.**

In Zambia, data were available for two time periods, 2004 and 2010. It was not possible to obtain data for more than one time period in any other country. Figure 1 shows the comparative CIs for the five countries, computed in this manner.

**Figure 2:**
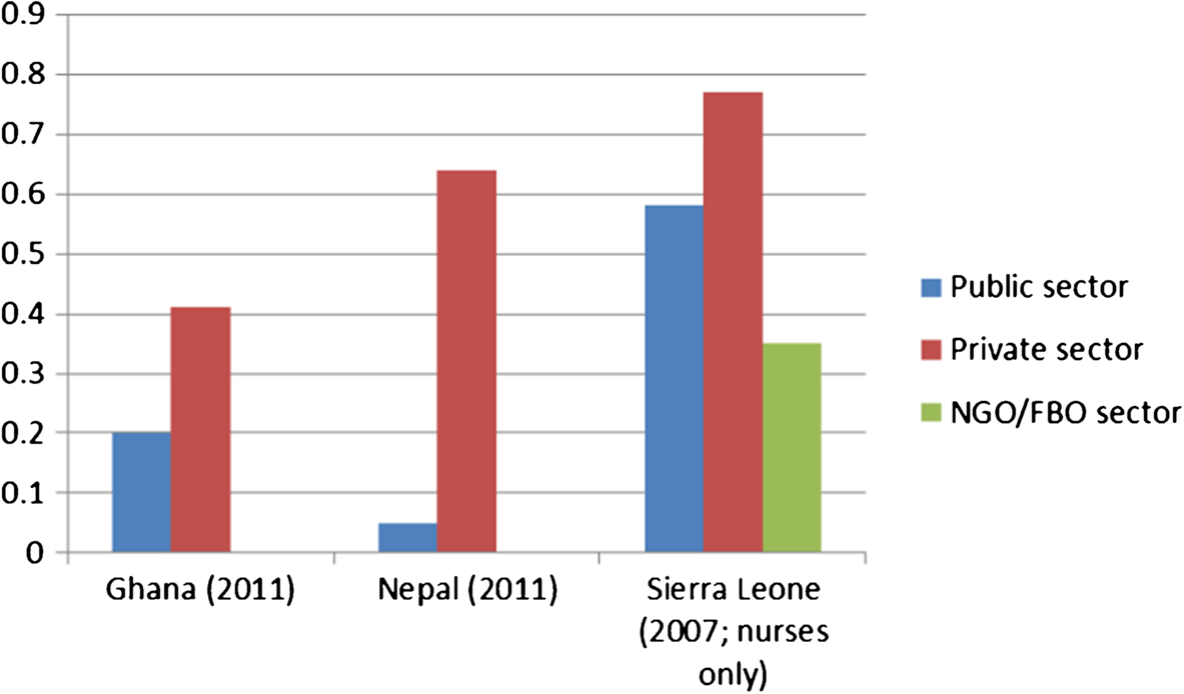
Concentration indices in public and private sectors.

The figure shows that in all countries but Sierra Leone, doctors are much more concentrated in densely populated (urban) areas than other cadres. In Sierra Leone, nurses and midwives are about equally concentrated in those areas, and no cadre provides cover for remoter rural areas. Overall, the concentration of doctors in urban areas is most pronounced in Nepal. Clinical officers in Zambia and Auxiliary Nurse Midwives (ANMs) in Nepal are spread almost equally across areas, in line with population numbers, suggesting the significant potential for such additional, non-traditional cadres to contribute to more equitable population coverage for RMNH services. However, in Zimbabwe it is nurse/midwives who make the most contribution to providing RMNH services in remoter rural areas. The number of clinical officers is very small and most of them are in Harare. Figure 2 compares public and private sector CIs for those countries for which that disaggregation was possible.

The figure shows that, according to those HRH data available, Nepal achieves fairly equitable distribution of health workers in its public sector: its high overall CIs (Figure 2) reflect the dominance of the private sector in the employment of health workers. In Ghana and Nepal, the private sector employs health workers predominantly in urban areas resulting in CIs considerably higher than for the public sector. The further disaggregation in Sierra Leone between NGO/FBO and other private sectors shows the importance of this distinction. In Sierra Leone, health workers in the NGO/FBO sector are most equitably distributed. Nevertheless, they are still highly concentrated in urban areas - more so than staff in the public sectors of Ghana and Nepal.

### Delivery workloads

Table 1 shows the workload in terms of the current numbers of deliveries per SBA and per doctor, and the number of births per SBA and per doctor in each country as a whole. This shows the actual workload, if evenly distributed among all health workers in the country and the full coverage workload, if all deliveries were attended by an SBA in a facility. In Zambia, the definition of an SBA is particularly difficult and we show these totals for both a 'narrow’ and a 'broad’ definition^e^.

**Table 1:** **Delivery workload for skilled birth attendants and doctors**: **actual rate of facility**-**based deliveries and full coverage** (**all births**)

	**Births per SBA**	**Births per doctor**	**Attended deliveries per SBA**	**Attended deliveries per doctor**
Ghana 2010/11	29	283	13	127
Nepal 2011	309	525	132	224
Sierra Leone	1202	1048	320	279
Zambia, narrow	185	1317	73	515
Zambia, broad	133		52	
Zimbabwe	18	475	12	313

*SBA* skilled birth attendant.

The WHO suggests that one doctor is required for around 1000 births^f^, to provide emergency intervention where there are complications before, during and after delivery, whereas a midwife can provide care for 175 births per year. On the basis of these assumptions, most countries do not have an absolute shortage of health workers relative to current levels of facility-based delivery, with the exception of Sierra Leone. This is not surprising, as the availability of health staff and the quality-of-care problems that can result from excess demand for their services, serve to constrain demand. In Sierra Leone, births with ANMs, Community Health Officers (CHOs) and nurses, as well as midwives and doctors, are counted as attended births although ANMs, CHOs and nurses do not meet the training requirements to be classified as SBAs [55]. Ghana and Zimbabwe even have sufficient staff to provide full coverage for facility-based SBAs. Other countries have some shortfalls in relation to ability to provide full coverage. Clearly, this includes Sierra Leone, which does not have sufficient staff to cope even with the current workload. Zambia does not have a sufficient number of doctors, nor marginally, skilled birth attendants, under the narrow definition. Nepal has sufficient doctors for full coverage but not SBAs.

However, the dominant problem restricting access to skilled birth attendance in a facility is distribution: both geographic distribution and distribution among the public and private sectors. In Ghana, all regions have sufficient SBAs and doctors to provide full coverage, although this may not be true at district level. The situation is similar in Zimbabwe for SBAs, although regional numbers suggest that most districts are likely to have sufficient SBAs to cope, even with full coverage. This is not the case for Zimbabwe, where doctors are insufficient to cover actual current workload in four out of ten regions, or to provide full coverage in seven out of ten regions. This compares to no more than 120 births and 100 deliveries per doctor in Harare and Bulawayo [51]. In Nepal, only two of five regions (the Central and Western Development Regions) have sufficient doctors for either current workload or full coverage, and whereas all regions have sufficient ANMs for the current workload, none have sufficient for full coverage. At the extreme, the Far Western Development Region has one doctor for every 7562 births and one ANM for every 517 births. Nearly all districts in Sierra Leone have insufficient staff to cope with current workload. At the extreme, Kailahun District has one midwife for every 17 415 births and one midwife for every 4627 current facility deliveries, explaining the use of under-skilled staff to play this role. In Zambia, 55 districts (76%) have insufficient doctors to provide coverage at a rate of one for every 1000 births; 13 (18%) have no doctors at all. In 36 districts (50%) there is insufficient staff for full coverage under the narrow definition of SBA and 8 districts (11%) have insufficient staff for actual levels of facility-based delivery under the broad definition.

We were able to break down staff and workload numbers by district and public and private sectors in Sierra Leone. Of our five case studies, Sierra Leone has some of the most extreme RMNH staff shortages according to the above analysis. The numbers cited above for Kailahun are unaffected by the public-private disaggregation, as there is no private sector of either type there. This is likely to apply in other contexts: those districts that are most under-served in general are those in which private sector presence is likely to be least. A better served district such as Western Area has a relatively manageable 119 actual deliveries per midwife. Excluding sources of private-sector care that number rises to 175, on the cusp of what is considered manageable and indicating that even Western region would require more public sector midwives to provide adequately skilled care to an increasing rate of utilization of SBAs in public facilities.

### Remuneration and terms and conditions

It is very difficult to compare terms and conditions. There are variations in entry-level qualifications required, length of training and other barriers to entry to the health professions, and some of the case studies show these to be in flux as attempts are made to cope with shortages by reducing such barriers. Conditions that are important to health workers cannot all be captured as a national-level phenomenon: the quality and security of accommodation available; the working conditions, including presence of utilities and availability of basic supplies to support effective work; and the sanitary and other infection prevention conditions cannot be effectively compared and summarized across countries.

We have attempted to compare public sector salaries for the main health professions involved in RMNH. This is complex for several reasons. First, health professions are defined slightly differently. For example, we have used the term, doctor, but attempted to capture the ranges of pay and allowances that apply to a health professional with a medical degree, operating as a general primary provider, excluding specialists operating at tertiary level from our calculations. However, pay scales often overlap between longer-serving general doctors and more junior specialists and an approximate cut-off was used in some cases. Enrolled and registered nurses are still separated categories in some countries (among our case studies, Sierra Leone). As previously discussed, midwives are not a separate category in all countries.

Second, comparisons of different currencies can be made in terms of purchasing-power parity. However estimates of the rate of translation of a currency to its international dollar value are not made continuously. Currently, the best available estimates are from 2009. For Zimbabwe, these relate to the pre-dollarized economy and cannot be used for our purposes. Zimbabwean estimates are consequently presented in US dollars.

Third, we are interested in the relative, as well as the absolute value of salaries. We have compared salary levels to measures of national income or national product per capita as a measure of this. However, income distributions may differ and good-quality data on income distribution in Africa are scarce. If health workers benchmark their standards of living against others within their society they deem professionally comparable, our analysis is unable to indicate what this comparison may indicate.

Table 2 shows the public-sector pay (salary midpoints) of health workers in international dollars^g^ and as a ratio to gross national income (GNI) per capita for the country^h^. In Zimbabwe the figures are for US dollars and gross domestic product (GDP) at current exchange rates^i^.

**Table 2:** **Public sector remuneration** (**salary midpoints incorporating allowances**) **in international dollars and as a ratio to GNI per capita** (**all current**: **December 2011**)

	**Value of salary and allowances in international dollars**			**Salary expressed as ratio to per capita gross national income**		
	**Doctor**^**a**^	**Nurse**	**Midwife**	**Doctor**	**Nurse**	**Midwife**
Ghana	3932	2171	2171	28.4	15.7	15.7
Nepal	4408	3851		43.7	38.2	
Sierra Leone	3179	429^c^	578^d^	46.0	6.2^c^	8.4^d^
Zambia	5346	2167		46.5	18.4	
Zimbabwe^b^	218	176		4.4	3.6	

^a^General medical doctor or closest equivalent available; ^b^Zimbabwe estimates are expressed in US dollars and as a ratio to per capita gross domestic product at current exchange rates; ^c^state-enrolled nurse; ^d^state-registered nurse and community midwife.

The data suggest that complaints about poor pay for most cadres in most countries would be unjustified, and in all cases health workers earn well above average rates of earning in their communities. Doctors in all countries but Zimbabwe appear to be among a rich elite earning 28- to 46-fold the average income. Nurses in Ghana, Nepal and Zambia are nearly as well paid, in the range of 15- to 38-fold. The recent pay award in Sierra Leone puts Sierra Leonean doctors into the same category, but leaves Sierra Leonean enrolled and registered nurses and midwives much less well paid. Zimbabwean health workers are more modestly paid than their counterparts in the other four countries. In those countries in which high salaries apply, reform of salary scales has been among the recent HRH policy innovations, suggesting a trend that other countries, and at least Zimbabwe among our case study countries, may have little choice but to follow.

As suggested above, the comparison with per capita GNP or GDP does not enable assessment of the relative remuneration in the sectors that health workers may deem comparable. It is difficult to obtain data for the top end of income distributions in African countries. Survey data are subject to large errors because of the small population earning at high levels and probable biases in the self-reporting of income among this population. The Zambia Living Conditions Monitoring Survey of 2004, for example, uses a cut-off of ZK 800 000 (approximately US$ 150 at current exchange rates) per month as its upper income threshold. Eighteen percent of Zambians stated an income of this level or above in 2004 and no further breakdowns of this figure are available. Doctors’ incomes have been estimated at more than 10-fold this cut-off, and nurses’ incomes at 3- to 4-fold.

Another possibility is that comparisons are not made locally but with international salary benchmarks. Given the increasing international mobility of health workers, it may be considered by governments setting salaries that health workers can only be retained if international salary levels can be matched. However, salaries so out of touch with the national economic capacity raise significant questions of sustainability, not only in a long-term future in which aid dependency is reduced, but also in an aid-supported future in which health worker numbers are significantly higher.

## Discussion

This study relied on secondary data and is constrained by the limited extent of those data. In particular, there were very few historic data that we were able to access, and we were generally unable to compare the situation of the health workforce before and after financing-policy change. Secondary data are also affected by well-known quality concerns that in the cases of individual datasets are difficult to assess. We benefited, however, from recent initiatives to strengthen HRH databases in several of the countries. Despite constraints in the data collection process, we believe the data we have used were the most up-to-date at the time of collection (2011) and those believed the best quality available in each country.

Of the four case-study countries that have removed or introduced exemptions for user fees for RMNH (in the fifth case-study country, Zimbabwe, no discrete policy change was introduced), only in Nepal is there clear evidence of positive impact on utilization without significant exception. In Ghana, better evidence is available in relation to the earlier maternal health exemption programme than the more recent inclusion of free maternal health services in the NHIS, although an evaluation of the NHIS exemption for pregnant women was due in 2012, according to Ministry of Health sources. It appears clear that utilization increased where free care was effectively available, but implementation difficulties, most notably under-funding of the programme, implied that effective free care (at least as judged by users) was not sustained, with the implication that higher rates of utilization also could not be sustained. In Zambia, fee removal was not specifically targeted at Maternal and Newborn Child Health (MNCH) services and there is conflicting evidence of the impact of fee removal on utilization. In Sierra Leone, data suggest an initial increase in outpatient visits for children under five years in the first year of the policy, but a gradual decline since then, and an overall fall in immunization levels, which may have been caused by factors external to the policy. These findings illustrate the importance of attending to the supply side, including human resource constraints, when seeking to support access to effective health care through financing policy change.

In Nepal and Zambia, there is some evidence that user-fee removal has particularly enhanced the utilization of poorer groups (Nepal) or areas (Zambia), although we have expressed some doubt about the Zambian analysis on this point. In other countries, it has not been possible to break down utilization change in this way.

The HRH situation in case-study countries is more variable than might have been expected. At national level, shortages of HR relative to the needs of RMNH services are not universal. However, in general, there are local shortages relative to need, either because of overall national level shortages, which are acute in Sierra Leone and more marginal in Zimbabwe, or because maldistribution creates local shortage where there is national sufficiency. The relative contribution of health workers in the private sector is difficult to measure. Although such workers represent capacity to deal with RMNH needs, they may be under-used to the extent that people are unable to access those health workers due to the financial barrier. In Nepal where the proportion of health staff in the private sector is highest, this issue is more important than it yet is in the African countries. However, economic and private sector growth in these countries implies that questions of access to private-sector health staff and their influence on the overall balance of need and HRH capacity will require a more sophisticated analysis.

Low salaries are not the general situation of health workers in the case-study countries, with salary levels for doctors in Nepal, Sierra Leone and Zambia, implying that they must be located at least in the top 2% of the income distribution, and in Ghana, the top 3 to 4%^j^. Other cadres, other than nurses in Nepal, are not quite so well paid. The situation in Sierra Leone for non-doctor health workers and for all health workers in Zimbabwe is more moderate, with pay levels at 3- to 9-fold per capita GNI/GDP.

The relatively high salary levels for at least some health workers suggest that their market position or collective bargaining power is strong. One explanation of this is the greatly increased level of international migration since the 1990s. This implies a global market for scarce medical skills in which some countries seem positioned to compete, although the sustainability of that level of competition is questionable both in the medium and long terms. Benchmarks are not available and the expectations of well-educated Africans and Asians, whose economies are characterized by high degrees of inequity in income distribution, are likely to be relatively high in comparison to national incomes per capita than in countries where education is less scarce. Given that only 2% of Zambians (for example, Zambia Living Conditions Monitoring Survey, 2004) are educated to degree level or above, it may be a reasonable expectation of those who are, that their incomes should locate them in the same elite.

Another key issue is the extent to which competence in skilled birth attendance is difficult to assess across the case-study countries. The research has relied on rules of thumb about who counts or does not count as an SBA. There are particular difficulties in this assessment in Zambia, where no separate category of midwife exists and where nurses are not all trained to an adequate level of competence in skilled birth attendance; and in Sierra Leone where Maternal Child Health (MCH) aides do not meet the international definition of SBA but are locally expected to play this role. Even health workers who have initially been provided with sufficient training but who are not highly motivated, have not subsequently practised in the role of SBA, or have not received sufficient in-service training since, will not in practice have the requisite level of skill. Hence, the capacity to scale up to 95% coverage of RMNH services is probably more limited than it appears.

This research highlights gaps in systematic and well planned coordination between financing policy and HR policy. In our case-study countries, there have been laudable attempts to plan for the impact of fee removal or reduction, and sometimes concomitant supportive change, even if not specifically responding to the needs of financing-policy change. The global literature review suggests that poor coordination is widespread. In some cases, such as in Niger and Zambia, measures were taken after problems associated with the removal of fees became evident. In the case of Zambia, of which we know more, the measures of compensation appeared to come too little and too late, sometimes not at all.

A number of countries that removed fees also increased health worker pay to some extent at around the same time, although it is not clear that this was directly in compensation of changes brought about by fees in all cases. In Sierra Leone, the two policy debates have been clearly linked and salaries were increased in preparation for the FHCP [56]. Such explicit linkage is not apparent in the other countries. In Zambia it is claimed that the user-fee removal policy came with no plan or budget to recruit and deploy health workers [57].

Rather like pay, in some cases additional recruitment was undertaken concurrent to fee reform, but it is not clear in Zambia or Ghana that this was carefully planned as part of a package of complementary policies. In contrast, Sierra Leone did plan increased recruitment as an element of the FHCP and this had been 'partially achieved’ at the time of a review in June 2010 in the sense that it was seen as contingent on the salary uplift.

Rather it appears that pay reform, recruitment activity and user-fee reform are among a plethora of interventions that are being introduced concurrently but with insufficient coordination. The literature on user fees is now quite clear on the need for associated measures, and the implications of the neglect of these are clear. In the first place, failure to coordinate undermines the impact of user-fee reform through what appear as implementation problems and result in the failure of policies to secure expected results or to sustain them. In the second place, user-fee reform may be exacerbating HR problems. The clearest case of this is the Zambian one. Ironically, the focus of user-fee removal on rural districts, intended to target access improvements in rural areas, has had a disproportionate effect on workloads in rural areas, which were already significantly greater than in urban areas. Worsening the relative conditions in rural areas is likely, other things being equal, to worsen the maldistribution of HRH and may result in rural access deteriorating. In Sierra Leone, loss of user-fee income has resulted in the loss of volunteer workers, who in practice depended on user fees for an income rather than constituting volunteers in the strict sense. This may explain the declining rate of immunization, as this appears to depend to some extent on such workers.

The difficulties of policy coordination are well-known and are not confined to low- and middle-income settings [56]. The specific set of policy process issues involved in user-fee removal have been analysed by Meessen *et al*. [58], who find that what they describe as 'good practice’ has more often than not been absent in the six African countries they review. One common feature they identify is a 'top-down’ and in many cases sudden and surprise move to remove charges that planners and policy makers at lower levels then struggle to adjust to. This may partly explain some of the problems in our case-study countries too.

The HRH situation also affects user-fee reform in the sense that there is some evidence among our case studies that staff who feel aggrieved because of a sense of overwork, underpay or deterioration in conditions, are more likely to undermine user-fee reform in the interests of maintaining the status quo. In all countries, there was evidence that services intended to be free were not always experienced as such by users, although to different extents. This problem was seen to be small in Sierra Leone, and to have reduced in Nepal, but indicates a clear link between the two areas of policy in this direction. At the extreme, informal fees can simply replace formal ones.

Linkage also operates in both directions through the medium of quality of care. User-fee removal can only be counted as successful to the extent that users recognize a better option in the reformed service, comparing both quality and price variables. Consideration of utilization as an indicator of the effectiveness of policy reform measures the direct and desired outcome, improved access, but also indicates the extent of users’ preferences for the reformed service [59]. The observation that initial increases in utilization are not sustained (of our case studies, most likely in Zambia) implies that neither measure of success is long lived. Health workers make perhaps the most critical contribution to quality of care and to whether any utilization gains following fee removal are sustained. Interpersonal aspects of quality of care - whether users are treated with dignity and respect and given the attention their problem requires - always rank highly in studies of the attributes of quality that matter to users, and are mainly under the control of health workers. Health workers also have influence on whether drugs and other supplies are available when required: they can conceal available stocks, and can use initiative to replace drugs that are out of stock at the time, for example. Aggrieved health workers who do not support user-fee removal because they have not been adequately compensated for the lost income and increased workload, are least likely to support the maintenance of quality in any of its dimensions.

Among the associated measures well-recognized in the existing literature is the need to ensure replacement of user-fee income where it is important at the local level. User-fee income has typically been used to provide bonuses to staff, employ additional contract staff and to support drug supply. All the case studies of user-fee removal or exemption except Nepal identified problems in either the failure to replace user-fee income or the inadequacy of the replacement in form or amount.

## Conclusions

The interconnectedness between user-fee policy and HRH situations has proved too difficult to assess with the existing evidence base. Many policies have been changing over the relevant period, some have changed explicitly in response to problems associated with financing policy change, others might have responded, but policy documents do not make this clear in their explanation of the policy rationale. Other relevant variables have also changed and we do not have evidence that would allow a full understanding of the state of a country’s health system in the absence of user-fee policy changes.

As is now well-recognized in the user-fee literature, coordination of health financing and HR policies is essential. This appears less well-recognized in the HR literature. In order to support (whole or partial) free health-care policies, investment needs to be made in pay and recruitment, and in particular to ensure that relative conditions of employment do not worsen for rural areas. Generalized pay increases in the context of an increasing imbalance of workloads in urban and rural areas may not be sufficient. Policy coordination proves an intractable problem in many settings, not only in low- and middle-income countries, but this does not mean that improvement is not possible within health policies, and across government and international stakeholders.

Replacement of user-fee income is only part of the solution to the management of the introduction of free health care. Human resources need specific attention in relation to recruitment and retention, and most of all, distribution. Policies focusing on incentives to attract health staff to under-served areas are weak across all case studies.

Demand-side financing approaches better replicate the positive aspects of the incentives embedded in user-fee systems, and appear to work well in Nepal. However, they may not work everywhere. They clearly can only work where reliable funding is maintained, as did not happen in Ghana. They may also require particular capacities of administrative systems, not always present, and it is noteworthy that the Nepal programme has been intensively supported with externally funded technical input, its management has not been integrated with general health system management and the challenge to achieve greater integration is now recognized. A careful analysis of the incentives embedded in alternative mechanisms of user-fee replacement is required everywhere for the most effective system in its context to be designed.

Policies need effective monitoring systems that focus on the realities of their implementation as well as their impacts. At present, the data required to monitor effectively are insufficient, despite some recent efforts to invest in this area.

## Endnotes

^a^[http://www.who.int/universal_health_coverage/en/];

^b^[http://www.icpdtaskforce.org/about/mission-vision.html];

^c^Service Availability and Readiness Assessment: see [REFD];

^d^[http://www.who.int/healthinfo/statistics/indbirthswithskilledhealthpersonnel/en/] (accessed 15 March 2013); ^e^the narrow definition includes only midwives, doctors and clinical officers. The broad definition includes midwives, doctors, clinical offers and nurses weighted for the percentage of obstetric workload in the total facility workload;

^f^The World Health Report, 2005 (p91) suggests that for a district with a birth rate of 30/1000, one full-time-equivalent doctor is required for 3600 births. Gabrysch *et al*. (2011) translates this into 1200 births per doctor on the basis of three doctors required to provide 24-hour cover. The WHO Making Pregnancy Safer model specifies 1000 births per doctor, and we apply this lower number, which also seems to allow for professional development days, leave and sick leave;

^g^PWT 7.0 Alan Heston, Robert Summers and Bettina Aten, Penn World Table Version 7.0, Center for International Comparisons of Production, Income and Prices at the University of Pennsylvania, May 2011. [http://pwt.econ.upenn.edu/php_site/pwt70/pwt70_form.php] (accessed 15 December 2011);

^h^[http://data.worldbank.org/indicator/NY.GNP.PCAP.PP.CD] (accessed 21 December 2011);

^i^[http://data.worldbank.org/indicator/NY.GDP.PCAP.CD] (accessed 15 December 2011);

^j^detailed income distribution data are not available but these estimates are based on the extreme assumption that those earning the given ratio of salaries to average GDP/GNI per capita capture virtually the whole GNI/GDP. More realistic assumptions rank the salary earner more highly still, relative to the rest of the population.

## Abbreviations

ANC: Antenatal care; ANM: Auxiliary nurse midwive; CHO: Community health officer; CI: Concentration index; DFID: Department of International Development (UK); FBO: Faith-based organization; FHCP: Free Health Care Policy; GDP: Gross domestic product; GNI: Gross national income; HDI: Human development index; HMIS: Health management information system; HR: Human resource; HRH: Human resources for health; ILO: International labour organisation; IPCD: International Conference on Population and Development; LCM: Living Conditions Measurement Survey; MCH: Maternal child health; MDG: Millennium Development Goals; MNCH: Maternal and newborn child health; MNH: Maternal and newborn health; NGO: Non governmental organization; NHIS: National Health Insurance Scheme; OPD: Out patients; PNC: Postnatal care; RMNH: Reproductive maternal newborn health; SARA: Service availability and readiness assessment; SBA: Skilled birth attendant; STD: Sexually transmitted disease; TBA: Traditional birth attendant; UHC: Universal health coverage; WHO: World Health Organization.

## Competing interests

The authors declare that they have no competing interests.

## Authors’ contributions

BM led the team, coordinated the research and was the lead author in drafting the paper. SW led the Ghana, Nepal and Zimbabwe case studies, contributed to cross-country analysis and commented on drafts of the paper. TE led the Zambia case study, led the cross-country analysis and commented on drafts of the paper. SF led the literature review work and commented on drafts of the paper. DN led the Sierra Leone case study and commented on drafts of the paper. TM contributed to the Nepal case study and commented on drafts of the paper. YC undertook fieldwork in Zimbabwe and commented on drafts of the paper. All authors read and approved the final manuscript.

## Authors’ information

BM is Professor and Director of the Institute for International Health and Development, Queen Margaret University, Edinburgh. Within the ReBuild research consortium on health systems development, she is a research director, and leading research on the effects of health-financing policy change on the household economies of the poor. She is a health economist specializing in health policy and health systems research in low- and middle-income countries. SW is Reader at the Institute for International Health and Development, Queen Margaret University, Edinburgh. Within the ReBUILD research consortium on health systems development she is leading research into health worker incentives in the post-conflict period. She is a health economist specializing in health financing in low- and middle-income countries, with a focus on reducing financial barriers (especially for maternal and child health services) and improving the delivery of services through effective purchasing and provider payment mechanisms. TE is Head of the Nuffield Centre for International Health and Development at the University of Leeds and Professor of International Health Systems Research. He is a health economist with experience of teaching, research and consultancy in more than 20 low- and middle-income countries. His work has focused mainly on health financing, particularly costing of services, informal health-care markets, demand-side mechanisms and health-system equity. SF is Senior Lecturer at the Institute for International Health and Development, Queen Margaret University, Edinburgh. Within the ReBUILD research consortium on health systems development she is leading research into aid effectiveness in fragile settings. She is a social scientist focusing on governance and health systems development in fragile states and other unstable settings. DN is Senior Lecturer in Economics at Aberdeen University. He is an international health economist, working principally in West Africa and South Asia, with a particular interest in the productivity costs of maternal ill-health. Within the ReBUILD research consortium, he is supporting research on innovative contracting arrangements for health-care provision in Cambodia and Sierra Leone. TM is a Senior Lecturer in Human Resource Management at the Liverpool School of Tropical Medicine, UK. He is the Co-research Director for the DFID-funded ReBUILD research consortium on health systems in post-conflict States and leads on the human resources theme. He is also Principal Investigator for PERFORM which is investigating ways of improving health workforce performance at district level in three African countries. He has carried out consultancies in HRH in many countries and is currently advising the Ministry of Health and Population in Nepal. YC is a social scientist and currently Senior Research fellow with the Biomedical Research and Training Institute Centre for International Health and Policy (BRTI-CIHP). He is leading ReBUILD research in Zimbabwe on health-worker incentives and rural deployment and posting of human resources for health. He has extensive experience in poverty assessment and was involved in the formulation and implementation of the Second Poverty Assessment Study Survey (Pass II) in Zimbabwe in 2003 and in the production of the provincial and national reports.
